# Evaluating Automated Tools for Lesion Detection on ^18^F Fluoroestradiol PET/CT Images and Assessment of Concordance with Standard-of-Care Imaging in Metastatic Breast Cancer

**DOI:** 10.1148/rycan.240253

**Published:** 2025-05-02

**Authors:** Renee Miller, Mark Battle, Kristen Wangerin, Daniel T. Huff, Amy J. Weisman, Song Chen, Timothy G. Perk, Gary A. Ulaner

**Affiliations:** ^1^GE HealthCare, Pollards Wood, Nightingales Lane, Chalfont Saint Giles HP8 4SP, United Kingdom; ^2^AIQ Solutions, Madison, Wis; ^3^Department of Nuclear Medicine, The First Hospital of China Medical University, Shenyang, China; ^4^Department of Molecular Imaging and Therapy, Hoag Family Cancer Institute, Irvine, Calif; ^5^Department of Radiology and Translational Genomics, University of Southern California, Los Angeles, Calif

**Keywords:** Molecular Imaging-Cancer, Neural Networks, PET/CT, Breast, Computer Applications-General (Informatics), Segmentation, ^18^F-FES PET, Metastatic Breast Cancer, Lesion Detection, Artificial Intelligence, Lesion Matching

## Abstract

**Purpose:**

To evaluate two automated tools for detecting lesions on fluorine 18 (^18^F) fluoroestradiol (FES) PET/CT images and assess concordance of ^18^F-FES PET/CT with standard diagnostic CT and/or ^18^F fluorodeoxyglucose (FDG) PET/CT in patients with breast cancer.

**Materials and Methods:**

This retrospective analysis of a prospective study included participants with breast cancer who underwent ^18^F-FES PET/CT examinations (*n* = 52), ^18^F-FDG PET/CT examinations (*n* = 13 of 52), and diagnostic CT examinations (*n* = 37 of 52). A convolutional neural network was trained for lesion detection using manually contoured lesions. Concordance in lesions labeled by a nuclear medicine physician between ^18^F-FES and ^18^F-FDG PET/CT and between ^18^F-FES PET/CT and diagnostic CT was assessed using an automated software medical device. Lesion detection performance was evaluated using sensitivity and false positives per participant. Wilcoxon tests were used for statistical comparisons.

**Results:**

The study included 52 participants. The lesion detection algorithm achieved a median sensitivity of 62% with 0 false positives per participant. Compared with sensitivity in overall lesion detection, the sensitivity was higher for detection of high-uptake lesions (maximum standardized uptake value > 1.5, *P* = .002) and similar for detection of large lesions (volume > 0.5 cm^3^, *P* = .15). The artificial intelligence (AI) lesion detection tool was combined with a standardized uptake value threshold to demonstrate a fully automated method of labeling patients as having FES-avid metastases. Additionally, automated concordance analysis showed that 17 of 25 participants (68%) had over half of the detected lesions across two modalities present on ^18^F-FES PET/CT images.

**Conclusion:**

An AI model was trained to detect lesions on ^18^F-FES PET/CT images and an automated concordance tool measured heterogeneity between ^18^F-FES PET/CT and standard-of-care imaging.

**Keywords:** Molecular Imaging-Cancer, Neural Networks, PET/CT, Breast, Computer Applications-General (Informatics), Segmentation, ^18^F-FES PET, Metastatic Breast Cancer, Lesion Detection, Artificial Intelligence, Lesion Matching

*Supplemental material is available for this article.*

Clinical Trials Identifier: NCT04883814

Published under a CC BY 4.0 license.

## Graphical abstract

**Figure:**
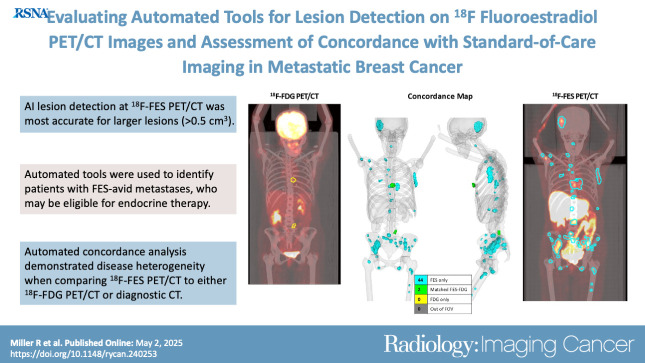

**Summary**

An artificial intelligence model was trained to detect lesions on fluorine 18 (^18^F)-fluoroestradiol (FES) PET/CT images and an automated concordance tool measured heterogeneity between ^18^F-FES PET/CT and standard-of-care imaging.

**Key Points**

■ An artificial intelligence (AI) model for lesion detection on 18 fluorine (^18^F) fluoroestradiol (FES) images had a median sensitivity of 62% for overall lesion detection, with a higher median sensitivity (90%) achieved for high-uptake lesions (maximum standardized uptake value [SUV] > 1.5, *P* = .002).
■ AI lesion detection plus an SUV threshold were combined to demonstrate a method for identifying patients with FES-avid metastases, who may be eligible for endocrine therapy.
■ Automated concordance analysis of lesions manually labeled on ^18^F-FES PET/CT, ^18^F-fluorodeoxyglucose PET/CT, and diagnostic CT images in 25 participants revealed that 17 participants (68%) had over half the lesions detected across all three modalities present on ^18^F-FES PET/CT images.

## Introduction

Breast cancer remains a significant health concern, accounting for nearly one-third of new cancer diagnoses in the United States as of 2023 (1). Although breast cancers exhibit heterogeneous receptor profiles, around seven in 10 express the estrogen receptor (ER) (2). In these tumors, ER signaling plays a key role in cell proliferation. Therefore, ER-targeting therapies are widely used as first-line treatment (3), and understanding the receptor status can help determine the best therapeutic approach. Typically, ER status is determined through the biopsy of a single lesion, chosen based on size, location, and accessibility. However, biopsies are subject to sampling error, and discordance in ER status between the primary and metastatic lesions has been observed in 6%–41% of patients (4). Additionally, the ER status within a single lesion may change over the course of disease progression (5,6). Therefore, the ER status of a single biopsied lesion at a single time point may not be representative of the ER status of all lesions over the course of the patient’s disease. Recent research suggests that immunohistochemistry assays to assess ER status identify the presence, not the activity, of ERs (7,8). Therefore, although a patient may present as ER-positive based on histology, the ERs may not be functional and may not respond to hormone-directed therapy (9).

Current standard-of-care imaging for patients with metastatic breast cancer includes follow-up CT imaging, using Response Evaluation Criteria in Solid Tumors to measure tumor response to therapy (10). In metastatic cases, fluorine 18 (^18^F) fluorodeoxyglucose (FDG) PET/CT imaging, highlighting regions of high metabolic activity, provides an important additional means of imaging patients to determine prognosis as well as measure treatment response (11). However, CT and ^18^F-FDG PET/CT each have limitations. In CT images, lesions in bone, a common metastatic site for metastatic breast cancer, are often not visible. Whereas with ^18^F-FDG PET/CT, physiologic uptake in the brain and other organs may obscure lesion detection locally. Additionally, breast cancers with invasive lobular carcinoma histology have low metabolic activity and may not be apparent on ^18^F-FDG PET/CT images (12,13). Finally, neither of these imaging methods reflects the current ER status of the lesions.

^18^F-fluorestradiol (FES) is a PET radiotracer that binds to ERs that are functional for estrogen binding, allowing for the detection of ER-positive lesions (14). The National Comprehensive Cancer Network guidelines recommend the use of ^18^F-FES PET/CT in certain cases during the systemic staging workup for patients with recurrent or metastatic breast cancer (14). Specifically, they state that ^18^F-FES PET/CT can be used to determine the overall ER status of a patient with metastatic breast cancer (15), which can be useful when lesions are difficult to biopsy or biopsy results are inconclusive (16). They state that ^18^F-FES PET/CT can confirm lesions that are equivocal with other standard-of-care imaging modalities (16).

One challenge for advanced imaging techniques is the time needed to fully identify and quantify lesions for subsequent interpretation. Manually identifying lesions on PET images can take 5–60 minutes per case, depending on the disease burden (17). Therefore, an automated lesion detection method could lead to substantial time savings and aid interpretation of ^18^F-FES PET scans.

Automated lesion detection can be challenging due to the heterogeneity of PET uptake across patients, healthy tissues, and scanner capabilities. For example, newer PET scanner models may have better resolution, especially for small lesions, compared with older scanner models. Significant work has been done in developing automated lesion detection on ^18^F-FDG PET/CT images in various cancer types including breast cancer. The majority of these methods use a U-Net architecture with a Dice loss, which has shown promising results (18–20). However, many previous methods emphasize whole-body disease burden voxel-level segmentation and quantification rather than individual lesion detection methods, which penalize region of interest (ROI)–level false positives and false negatives (21,22). Examples of deep learning architectures that emphasize lesion detection include Mask R-CNN (23), Retina Net (24), and Retina U-Net (25). The Retina U-Net architecture balances both the Dice segmentation loss and individual lesion detection and has shown promising results in PET/CT lesion detection (26–28).

This work presents two automated tools for assessing ^18^F-FES PET/CT images and explores their utility for the clinical workflow. Specifically, a lesion detection algorithm was trained and assessed for its ability to provide automatic detection of FES-avid lesions, thereby assisting in determining whether a patient is suitable for endocrine therapy. Additionally, an automated tool using rule-based methods was used to assess concordance between lesions identified by a nuclear medicine physician on ^18^F-FES PET/CT scans and those identified on either diagnostic CT or ^18^F-FDG PET/CT scans. We aimed to demonstrate the feasibility of automated tools in augmenting visual reads of ^18^F-FES PET/CT scans. The downstream aim is to provide a tool that incorporates the two algorithms, automated lesion detection and concordance analysis, in a single end-to-end solution in which the lesions automatically detected from the ^18^F-FES PET/CT images are fed directly into the concordance analysis tool. However, this initial work presents results from the two tools separately and represents a proof of concept of each step.

## Materials and Methods

### Study Design and Participants

This secondary analysis included 52 participants with breast cancer who underwent ^18^F-FES PET/CT at Hoag Memorial Hospital Presbyterian as part of a prospective trial comparing ^18^F-FES PET/CT to standard-of-care ^18^F-FDG PET/CT or CT imaging for detecting breast cancer metastasis and recurrence (ClinicalTrials.gov identifier: NCT04883814 [29]). The study was performed after approval by the Western Institutional Review Board Copernicus Group and adheres to the Standards for Reporting of Diagnostic Accuracy guideline. GE HealthCare provided financial support as an investigator sponsored trial. For the current work, authors affiliated with GE HealthCare (R.M., M.B., K.W.) did not control data inclusion or exclusion.

Female patients aged 18 years or older with ER-positive breast cancer confirmed with immunohistochemistry were eligible for inclusion after providing written informed consent. Participants who were pregnant or lactating, unwilling to provide written informed consent, male patients, or patients currently using tamoxifen or fulvestrant were excluded from the study. Full study details including a flowchart of participant inclusion and exclusion criteria can be found in the study by Ulaner et al (29). Thirty-six of the 52 participants underwent diagnostic CT as well as ^18^F-FES PET/CT; 14 underwent ^18^F-FDG PET/CT and ^18^F-FES PET/CT; and one underwent all three imaging modalities (standard-of-care CT, ^18^F-FES PET/CT, and ^18^F-FDG PET/CT).

Participants were part of one of two study arms. The first arm included participants with newly diagnosed breast cancer at stage 2 or 3 who were undergoing imaging to investigate suspected distant metastases. The second arm included participants who were undergoing imaging to detect suspected recurrence. Therefore, due to the nature of the inclusion criteria, none of the participants in the first arm had undergone any prior treatment, whereas all participants in the second arm had undergone at least one prior round of treatment.

### Imaging Protocol

Participants underwent ^18^F-FES PET/CT imaging within 14 days of the standard-of-care imaging (CT or ^18^F-FDG PET/CT). Approximately 5 miCi of FES was administered intravenously before PET/CT imaging. Standard-of-care CT and ^18^F-FDG PET/CT were performed according to standard clinical protocol.

### Lesion Labeling

Lesions were manually contoured on the ^18^F-FES PET/CT images by an experienced nuclear medicine physician (S.C., with 13 years of experience) using both the ^18^F-FES PET image and the associated CT image acquired on the dedicated PET/CT scanner for PET attenuation correction. Lesions were identified based on the following abnormal patterns: *(a)* regions with high uptake on ^18^F-FES PET images and abnormal patterns on attenuation CT images, *(b)* regions with high uptake on the PET images that had normal appearance on the attenuation CT images, and *(c)* regions that had abnormal appearance on the attenuation CT images with no FES avidity. For this investigation, lesions in all classes were treated as confirmed malignancies.

Additionally, lesions were manually contoured on the standard-of-care CT and/or ^18^F-FDG PET/CT images by the same nuclear medicine physician for a subset of 25 participants to assess concordance between ^18^F-FES PET/CT and standard-of-care images. The subset of participants was randomly chosen from participants having disease on one or both imaging modalities. A total of 13 CT images and 13 ^18^F-FDG PET/CT images were contoured, with one participant having undergone all three imaging methods (^18^F-FES PET, ^18^F-FDG PET, and CT).

To assess interreader variability of ^18^F-FES PET/CT contouring and establish a benchmark for automated lesion detection methods, the ^18^F-FES PET/CT images were contoured by a second reader (Laurence Vass, with 12 years of experience in nuclear medicine contouring).

### Automated Lesion Detection

A deep learning model with a Retina U-Net architecture (25) was trained for lesion detection using fivefold cross-validation with the 52 ^18^F-FES PET/CT examinations. Thus, five models were trained in total with 41 or 42 examinations in the development set, and 10 or 11 examinations in the testing set, with all participant images included in the testing set exactly once. Due to the limited number of examinations in the development set, a small number of examinations (two) was chosen for monitoring and learning rate scheduling. The remainder of the examinations were used for training.

The Retina U-Net architecture and training regimen was implemented because it combines loss from both a segmentation arm and a lesion detection arm, thus weighting more equally the loss of small lesions compared with segmentation-only networks such as the U-Net. Retina U-Net was implemented as in Jaeger et al (25) with the following configuration changes: Two input channels were used, one for the ^18^F-FES PET and one for the attenuation correction CT image, which where both were resampled to the same grid size of 2.0 × 2.0 × 2.0 mm, chosen to balance the low resolution of the PET image and the high resolution of the CT image. Patches of size 128 × 128 × 128 were extracted from the training examinations using class balancing to ensure at least 20% of the sampled patches contained a lesion that was extracted from the PET/CT examinations on the fly. Data augmentations of random rotations and scaling were applied during training. A batch size of two was used for training, which was performed from scratch after random initialization for 250 epochs, with an epoch being defined as 1200 train batches. The loss was optimized using an Adam optimizer with dynamic learning rate scheduling, decreasing the learning rate by a factor of one-fourth upon plateau of the average precision. The model weights from the five epochs with the highest average precision in the monitoring dataset were used for inference, taking the average of the probabilities of each. The final output was converted to a binary mask using the .5 probability cutoff point.

Model performance was characterized using the free-response receiver operating characteristic paradigm, where performance is described by the sensitivity (the proportion of physician-identified ROIs also detected by the convolutional neural network [CNN]) and by the number of false positives per image (the number of ROIs detected by the CNN that were not identified by the physician). This approach is consistent with assessments of automated CNN lesion detection, where a concept of a "true negative lesion" does not exist and voxel-level specificity is not appropriate because the majority of voxels within the image are true negatives (21). Any predicted ROI within 10 mm of a reference standard lesion was classified as a true positive, and any two false positives within 10 mm of one another were counted as a single false positive. Note that, due to the low resolution of PET imaging, this buffer is approximately 2 voxels.

### Automated Identification of FES-avid Metastases

The presence of FES-avid metastases in participants was automatically determined using the AI-labeled lesions in the ^18^F-FES PET/CT images and the maximum SUV (SUV_max_) thresholds described in the study by van Geel et al (15). In brief, participants having at least one AI-labeled lesion with an SUV_max_ greater than 2.5 were labeled as having FES-avid metastases; participants having at least one lesion with an SUV_max_ between 1.5 and 2.5 were labeled as "likely FES-avid metastases"; and participants with no lesions having an SUV_max_ above 1.5 were labeled as having no FES-avid metastases. This was also completed using the manually drawn contours from the nuclear medicine physician.

### Automated Concordance Analysis

Images were analyzed using TRAQinform IQ software (AIQ Solutions) to automatically quantify physician-delineated lesion ROIs on the ^18^F-FES PET/CT, ^18^F-FDG PET/CT, and standard-of-care diagnostic CT scans and to match ROIs between different modalities (17,30). TRAQinform IQ classifies each lesion based on its presence at either or both examinations: ^18^F-FES PET/CT only, standard-of-care only (^18^F-FDG PET/CT or diagnostic CT), or both ^18^F-FES PET/CT and standard-of-care examination. TRAQinform IQ is a software medical device that performs comprehensive ROI-level estimation of anatomic and functional change derived from augmentative software analysis of multiple CT or PET/CT scans including total and individual changes of tracer uptake, radiodensity volumes, and heterogeneity of change, with interpretation.

### Statistical Analysis

For the lesion detection algorithm, performance was quantified overall (across all 52 participants) for all lesions. Additionally, performance was assessed in only ROIs with SUV_max_ greater than 1.5 and separately with volume larger than 0.5 cm^3^. Differences in sensitivity were assessed using a paired Wilcoxon test with a level of *P* value less than .05 determining significance. Reference standard lesions and predicted ROI were classified based on their location using TRAQinform IQ’s organ and region segmentation tool (AIQ Solutions). The locations extracted for this analysis were the entire skeleton and the chest region (includes breast). For the concordance analysis, the number and percentage of lesions in each category were quantified for each patient. This was completed for a subset of 25 participants: 13 ^18^F-FES to ^18^F-FDG comparisons and 13 ^18^F-FES to diagnostic CT comparisons, with one patient included in both comparisons as they underwent all three imaging acquisitions. Statistical analysis was performed using Python version 3.9 and R version 4.4.0.

## Results

### Participant Characteristics

A summary of patient information, including the type of imaging performed, subtype, primary tumor grade, hormone receptor status from histology, and cancer stage, is shown in Table 1. Number of patients excluded, mean ages of the patients, and further study details can be found in the study by Ulaner et al (29).

**Table 1:**
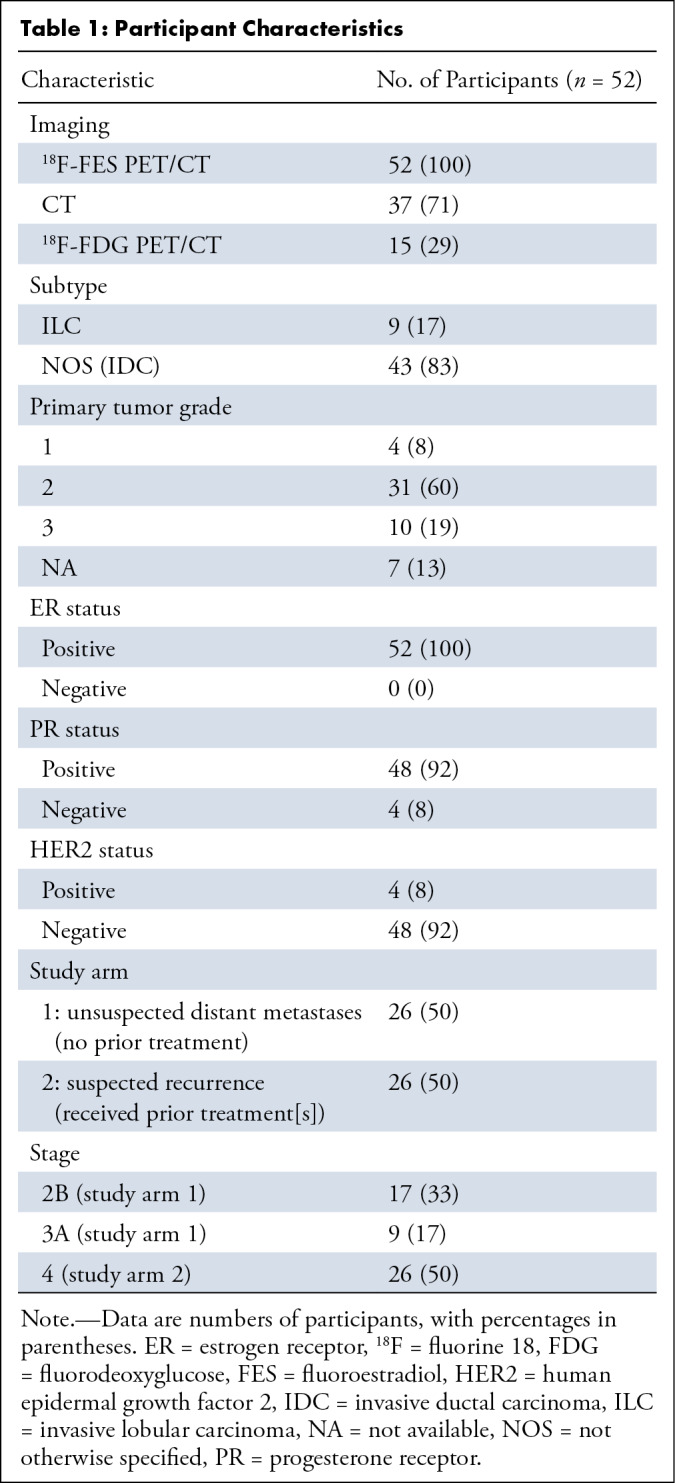
Participant Characteristics

### Automated Lesion Detection and Quantification

Across the 52 participants, the algorithm detected a total of 752 lesions on the ^18^F-FES PET scans (median per patient: four; range, 0–189). Of these, 502 of 752 (66.8%) had an SUV_max_ greater than 1.5 g/mL (two; range, 0–188), and 443 of 752 (58.9%) had volume greater than 0.5 cm^3 ^(three; range, 0–138).

**Figure 1:**
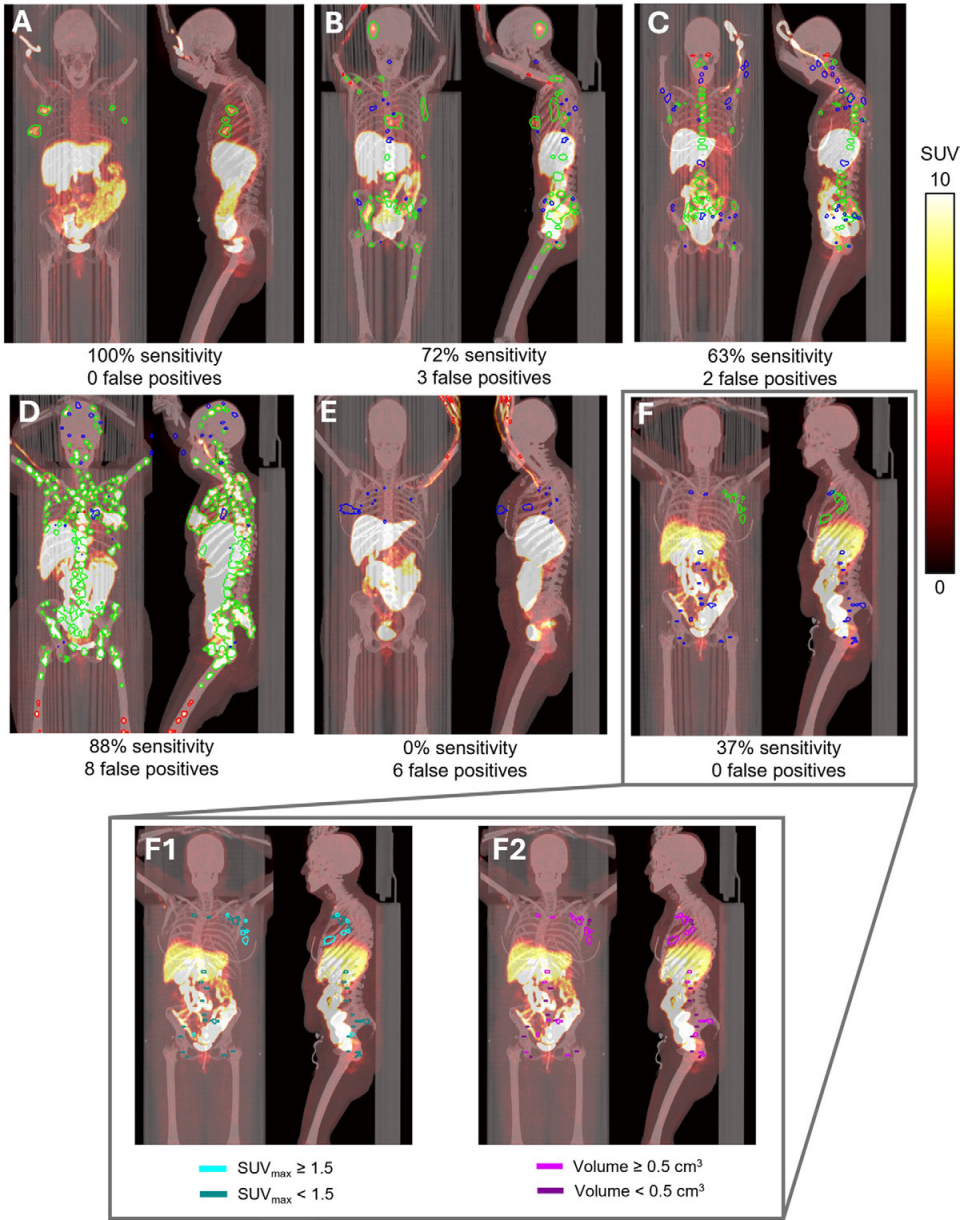
Example scans in women with breast cancer of automated lesion identification from fluorine 18 (^18^F)-fluoroestradiol (FES) PET/CT highlighting true positives (green), false positives (red), and false negatives (blue). **(A)** All four lesions labeled in the reference standard annotation were detected by the model (100% sensitivity). **(B)** Thirty-three of the 46 lesions were detected by the model (72% sensitivity), whereas 13 lesions were not (false negatives). Additionally, three regions were labeled by the model that were not labeled by the expert reader (false positives). These false positives were located in the peripheral vein where the tracer was injected. **(C)** The model detected 46 of 72 lesions (63% sensitivity) but found two additional regions (false positives). **(D)** One case with relatively high sensitivity (finding 167 of 189 lesions, 88%) yet with eight false positives. Most missed lesions from this case were in the head and upper extremities. The false positives were in the right and left femur and could indicate lesions missed by the human reader. **(E)** The case shows numerous false positives in the peripheral vein where the tracer was injected. At the same time, the model failed to detect all lesions in the chest. **(F)** The model only detected 10 of 29 total lesions (34% sensitivity) labeled in the reference standard. The same participant is shown in panel F, highlighting lesions that had **(F1)** low tracer uptake (maximum standardized uptake value [SUV_max_] < 1.5) and **(F2)** small lesions (volume < 0.5 cm^3^).

Figure 1 illustrates six example ^18^F-FES PET/CT images with the automated detection results showing cases with above or at the median accuracy of the model in the top row, and the bottom row highlights cases that were less accurate (low sensitivity or a large number of false-positive regions). Additionally, Figure 1 (1F1, 1F2) illustrates, in one patient, the accuracy of the model to detect lesions with either low uptake (Fig 1F1) or small volumes (Fig 1F2). It can be noticed that the model primarily detected larger lesions (volume > 0.5 cm^3^) and those with higher uptake (SUV_max_ > 1.5).

**Figure 2:**
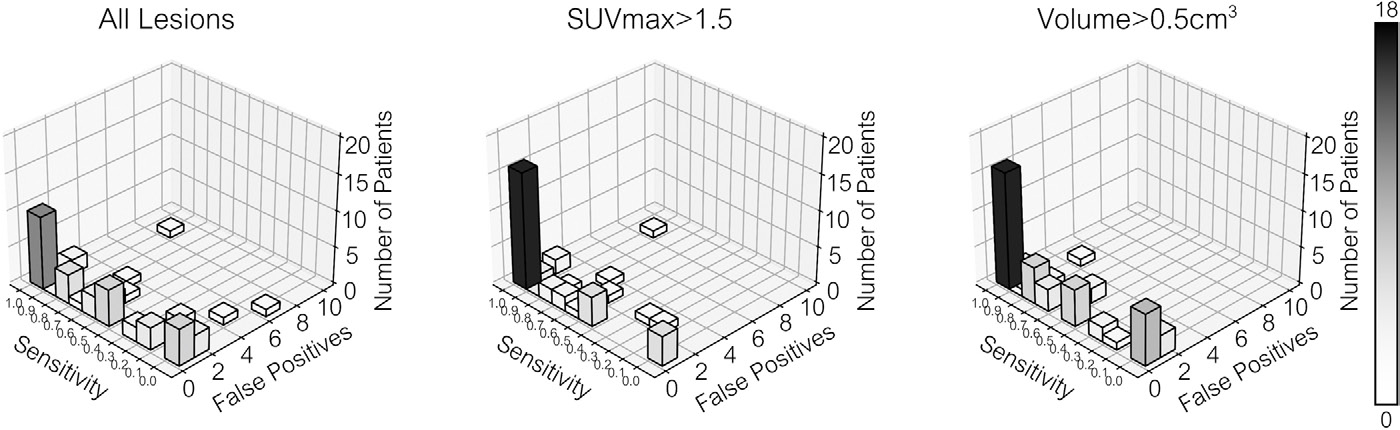
Accuracy of the automated lesion detection algorithm. A three-dimensional bar plot is shown to demonstrate that the model had the same performance in multiple patients. When including all lesions in the results, the median performance was 62% sensitivity with 0 false positives per patient, and the algorithm had perfect performance (100% sensitivity and no false positives) in 10 of 52 participants. However, if only lesions with high uptake (maximum standardized uptake value [SUV_max_] > 1.5) were included, the median performance was 90% sensitivity with 0 false positives per patient, and the model performed perfectly in 16 of 52 participants. Finally, if small lesions were removed from the analysis (with a volume < 0.5 cm^3^), the median performance was 80% sensitivity with 0 false positives per patient, and the algorithm performed perfectly in 16 of 52 participants.

Figure 2 shows quantified sensitivity and false positives across all 52 participants and characterizes the performance by SUV_max_ and volume of the regions. A table listing the model performance for each individual patient can be found in Table S1. The median performance in all lesions was 62% sensitivity with 0 false positives per patient. The AI model demonstrated higher sensitivity (median of 90%) in detection of high-uptake lesions (SUV_max_ > 1.5, *P* = .002) compared with overall lesion detection. However, there was no evidence of a difference in sensitivity for detection of large lesions (volume > 0.5 cm^3^, median sensitivity of 80%, *P* = .15) compared with overall lesions. Table 2 illustrates the accuracy of the model for the whole body, chest soft tissues only, and skeleton only, with IQRs. The sensitivity was highest for lesions in the chest only (including breast lesions) and lowest for lesions in the skeleton.

**Table 2:**
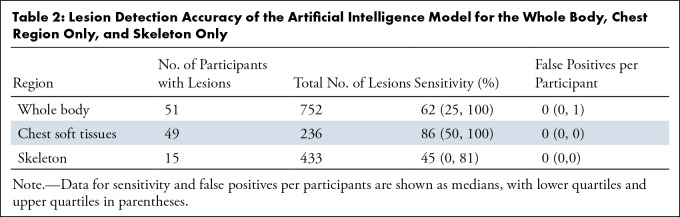
Lesion Detection Accuracy of the Artificial Intelligence Model for the Whole Body, Chest Region Only, and Skeleton Only

Across the 52 participants, the second FES reader (Laurence Vass) contoured a total of 442 (median per patient: one; range, 0–195) lesions. Of these, 434 of 442 (98%) had SUV_max_ greater than 1.5 g/mL (one; range, 0–192), and 376 of 442 (85%) had volume greater than 0.5 cm^3^ (one; range, 0–166). When comparing this reader to the original FES reader (S.C.), the second reader had a median sensitivity of 42% with 0 false positives per patient. In lesions with SUV_max_ greater than 1.5, the median sensitivity of the second reader (Laurence Vass) was 76% with 0 false positives per patient, and in lesions with a volume greater than 0.5 cm^3^, the median sensitivity of the second reader (Laurence Vass) was 67% with 0 false positives per patient.

### Automated Identification of FES-avid Metastases

Table 3 illustrates which participants, at the time of imaging, have no FES-avid metastases, likely have FES-avid metastases, or have FES-avid metastases, using AI and manually labeled lesions and SUV thresholds defined in the study by van Geel et al (15). Of the 11 participants labeled as having no FES-avid metastases using manually labeled lesions, 10 underwent resection of the primary tumor (either lumpectomy or full mastectomy) before ^18^F-FES PET/CT imaging. Similarly, nine of 10 participants labeled as having no FES-avid metastases from the AI-labeled lesions underwent resection of the primary tumor before imaging. In these two cases that did not include resection of the primary tumor, the primary lesion had responded to other therapy and was not apparent at ^18^F-FES PET (SUV_max_ < 1.5) at the time of imaging.

**Table 3:**
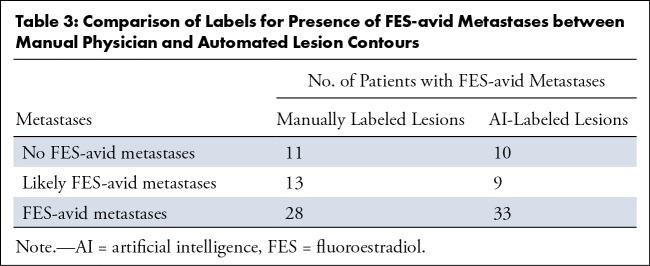
Comparison of Labels for Presence of FES-avid Metastases between Manual Physician and Automated Lesion Contours

**Figure 3:**
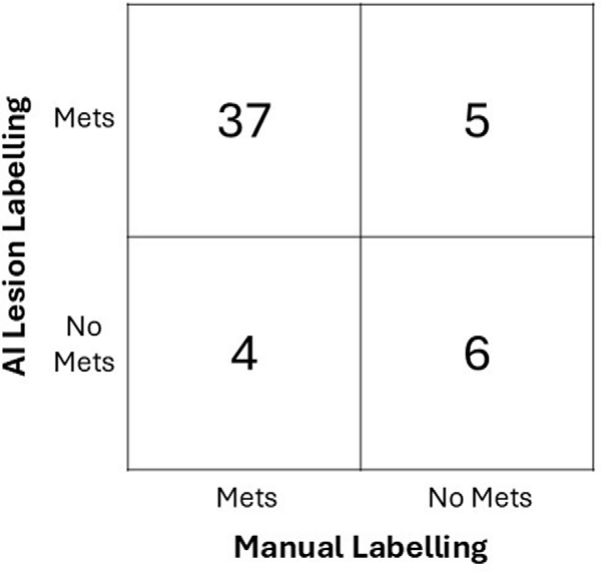
Confusion matrix illustrates accuracy of the AI method for labeling patients as either having (or likely having) breast cancer metastases or not having metastases. Met = metastases.

The AI tool for detecting lesions, when used in conjunction with the thresholds defined in the study by van Geel et al (15), was shown to have a sensitivity of 90% (37 of 41, 95% CI: 76, 97) to detect which participants had FES-avid metastases (including FES-avid metastases and likely FES-avid metastases), and required no manual input. However, the automated tool erroneously labeled four participants as not having FES-avid metastases when the automated tool failed to detect lesions (three of four had low uptake: SUV_max_ < 2.5). Likewise, due to the presence of false positive lesions in some participants, five participants were falsely labeled as having FES-avid metastases, leading to an overall specificity of 55% (six of 11, 95% CI: 25, 82). See Figure 3 for the full confusion matrix.

### Automated Concordance Analysis

**Figure 4:**
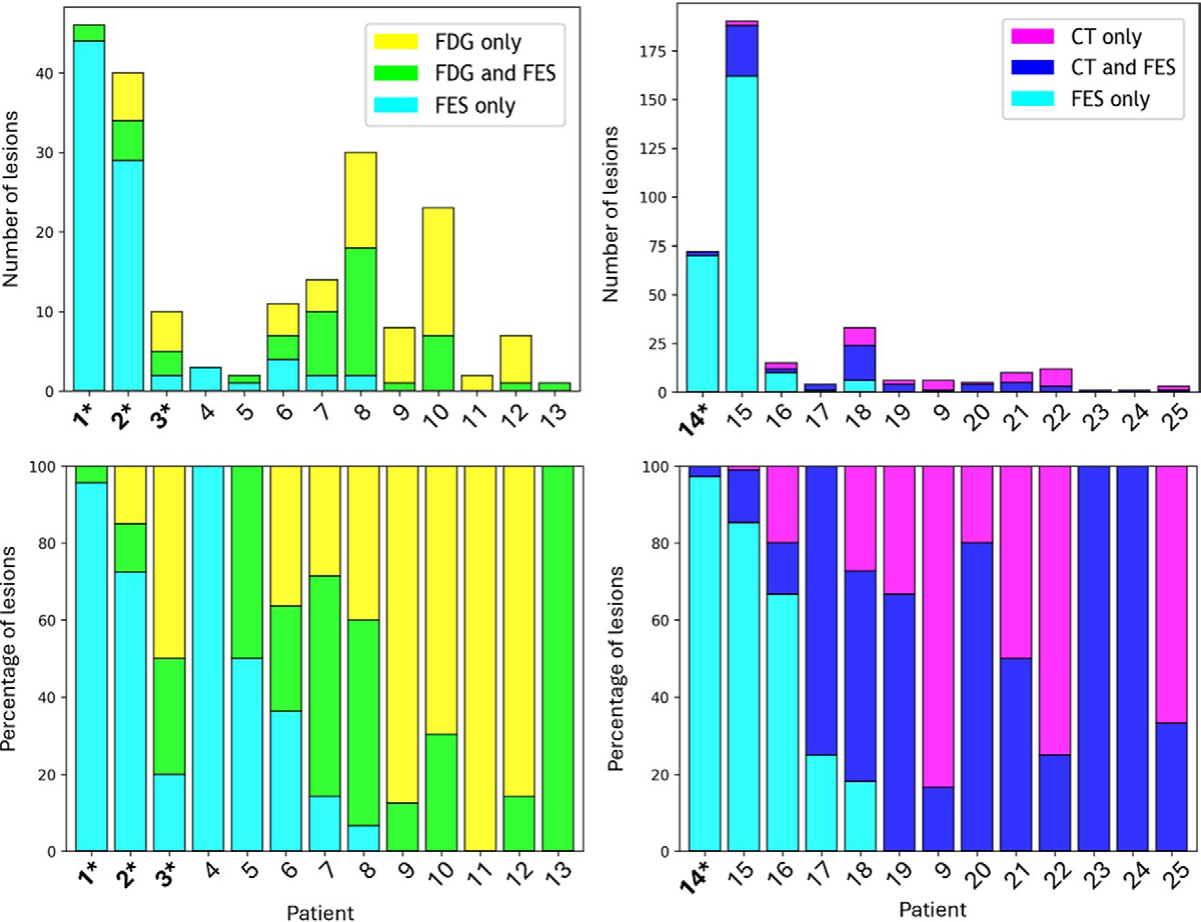
Overall results of the automated concordance analysis, showing the number (top row) and percentage (bottom row) of lesions manually detected by a nuclear medicine physician with each imaging modality. Results comparing fluorine 18 (^18^F)-fluoroestradiol (FES) PET/CT to ^18^F-fluorodeoxyglucose (FDG) PET/CT are shown in the left two plots, and results comparing ^18^F-FES PET/CT to standard-of-care CT are shown in the right two. Participants with invasive lobular carcinoma are indicated by bolding and an asterisk.

**Figure 5:**
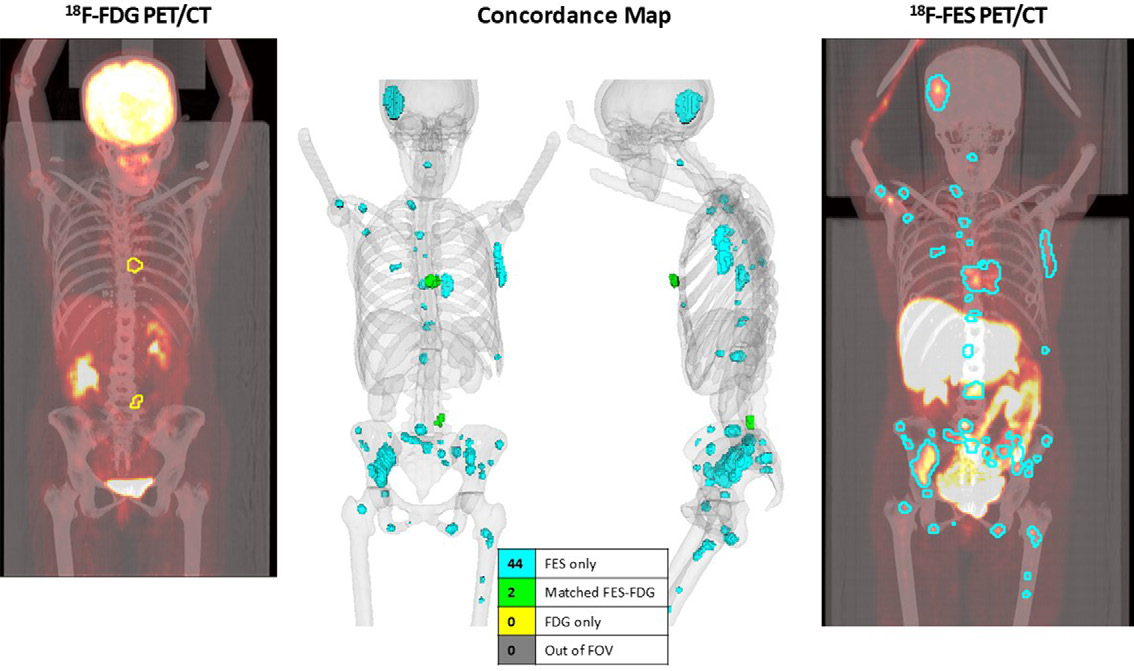
In one selected participant with invasive lobular carcinoma, the automated concordance tool (center) highlights two lesions (green), which were found in both the (left) fluorine 18 (^18^F)-fluorodeoxyglucose (FDG) PET/CT and (right) ^18^F-fluoroestradiol (FES) PET/CT images, whereas 44 lesions (light blue) were identified in only the ^18^F-FES PET/CT image and none were found in only the ^18^F-FDG PET/CT image.

**Figure 6:**
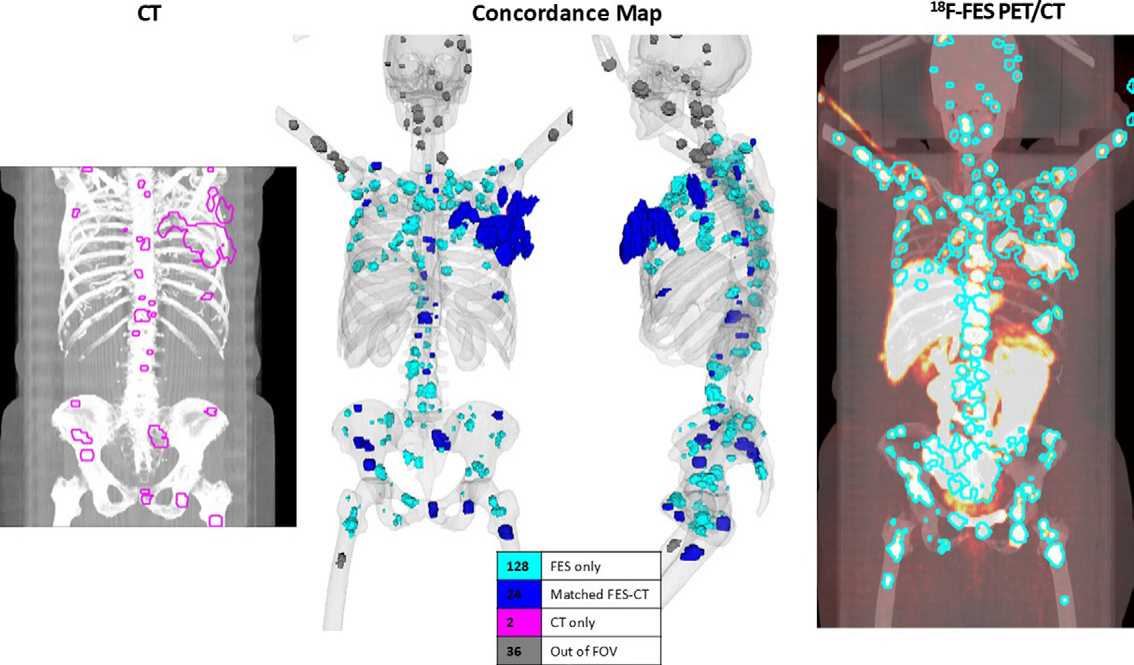
In one selected participant, the automated concordance tool (center) highlights 24 lesions (dark blue) that were found in both the standard-of-care CT image (left) and fluorine 18 (^18^F)-fluorodeoxyglucose (FES) PET/CT image (right); 128 lesions (light blue) were identified with only the ^18^F-FES PET/CT, and two lesions were found with only the standard-of-care CT. Seven lesions were outside of the CT field of view (gray).

Overall findings of the concordance analysis for the subset of 25 participants for both ^18^F-FDG PET/CT and standard-of-care diagnostic CT are shown in Figure 4. Disease heterogeneity, defined as having at least one lesion that appeared in one but not both modalities, was present in 12 of 13 cases comparing ^18^F-FES PET/CT with ^18^F-FDG PET/CT and in 11 of 13 cases comparing ^18^F-FES PET/CT with diagnostic CT. Across all analyzed participants, only one of 25 (4%) had no lesions detected with ^18^F-FES PET/CT. In 17 of 25 participants (68%), over half of the detected lesions were present at ^18^F-FES PET/CT. Similarly, in 18 of 25 participants (72%), over half of the detected lesions were present on standard-of-care images (^18^F-FDG PET/CT or diagnostic CT). Six of 25 participants (24%) had the majority of their lesions detected with ^18^F-FES PET/CT only. Four participants chosen randomly for the concordance analysis had invasive lobular carcinoma (Fig 4). Three of these four participants showed a large proportion of lesions (>70%) only detected at ^18^F-FES PET/CT. Figures 5 and 6 show concordance maps for individual participants comparing ^18^F-FDG PET/CT to ^18^F-FES PET/CT (Fig 5) and a diagnostic CT to ^18^F-FES PET/CT (Fig 6). In particular, Figure 5 highlights one participant with invasive lobular carcinoma in which only two lesions were FDG-avid, whereas 46 lesions were FES-avid.

## Discussion

This study presented two automated image analysis tools and their applications and clinical use cases for managing patients with metastatic breast cancer using ^18^F-FES PET/CT imaging. First, exploratory results in a small dataset showed a lesion detection algorithm was able to achieve excellent performance in detecting FES-avid likely malignant lesions, with a median sensitivity of 90% in lesions with an SUV_max_ greater than 1.5. Second, an automated concordance analysis of reference standard lesions on ^18^F-FES PET/CT and standard-of-care images was performed, highlighting the anatomic and functional and molecular heterogeneity of metastatic breast cancer across lesions and participants with 12 of 13 cases displaying evidence of disease heterogeneity. This automated approach, when expanded and validated in larger datasets, has the potential to assess the presence of functional ERs within all lesions in a patient, which is currently infeasible manually due to the potentially high numbers of lesions that can be present in metastatic patients.

To the authors’ knowledge, this article presents the first AI model for lesion detection in ^18^F-FES PET/CT images. Although there are several previous studies applying AI methods for automated lesion detection in ^18^F-FDG PET/CT scans of patients with metastatic breast cancer, often research limits the scope of lesion detection, for example, only aiming to detect bone lesions (31) or lesions well above the PET background (18). In Moreau et al (19), an nnU-Net model was trained with 60 ^18^F-FDG PET/CT images to detect metastatic breast cancer lesions. The study presents an average lesion detection sensitivity of 72% with baseline images and 43% with follow-up images, though there is no description of whether the manual reference standard segmentations included low-uptake PET lesions or if the sensitivity was higher in lesions above a given SUV_max_ cutoff. This performance is similar to the detection sensitivity reported in the current study (median of 62%), and the Retina U-Net method achieved a median sensitivity of 90% in lesions with an SUV_max_ greater than 1.5 g/mL. Because 502 of 752 (67%) and 443 of 752 (59%) of the lesions manually segmented had an SUV_max_ greater than 1.5 g/mL or a volume greater than 0.5 cm^3^, respectively, the reduced sensitivity of detecting low-uptake small lesions may be attributed to data imbalance, wider heterogeneity in the appearance of these lesions, or more subtle imaging abnormalities compared with high-uptake larger lesions. For example, low-uptake lesions may be more difficult for the model to learn to detect due to having an abnormal pattern on the CT image but a normal pattern on the ^18^F-FES PET image. Lesion detection sensitivity was higher in the chest soft tissue region compared with the skeleton, although sample size limitations prevent statistical testing. In fact, 49 of 52 (94%) participants had lesions in the chest, whereas only 15 of 52 (29%) participants had lesions in the skeleton. Model sensitivity for detecting small or low-uptake lesions may be improved with a larger training dataset combined with class balancing techniques. Furthermore, a larger training dataset may reduce the number of false positives related to phenomena common to many images, such as uptake at the injection site.

The automated concordance analysis, which was applied to the reference standard lesion contours for all image modalities, illustrates the utility of ^18^F-FES PET/CT in several clinical applications for patients with metastatic breast cancer. First, results showed that six of the subset of participants had the majority of lesions detected only at ^18^F-FES PET/CT. In these participants, and in others with lesions detected only at ^18^F-FES PET/CT, a change in patient management may be appropriate due to this finding. It is common to find equivocal lesions with standard-of-care imaging modalities such as diagnostic CT and ^18^F-FDG PET/CT due to their high sensitivity and low specificity. More specifically, in patients with invasive lobular carcinoma, tumors often have low metabolic activity and therefore do not have high uptake on ^18^F-FDG PET/CT scans (12,32). Therefore, any ER-positive lesions will be detected at ^18^F-FES PET/CT examinations but not at ^18^F-FDG PET/CT for this patient group. Although lesions may be visible in the attenuation correction portion of the PET/CT, this was not used in the concordance analysis because the quality of the attenuation CT varies greatly depending on the acquisition protocol and parameters. Therefore, when performing the concordance analysis comparing ^18^F-FES to CT, only the diagnostic CT was used, despite having a smaller field of view than that of PET/CT.

Interreader variability of ^18^F-FES PET/CT was poor in this patient population; however, this is largely due to the second reader contouring predominantly high uptake (SUV_max_ > 1.5) lesions. In fact, 502 of 752 (67%) of lesions contoured by the first reader (S.C.) had an SUV_max_ greater than 1.5, whereas 434 of 442 (98%) of the lesions contoured by the second reader (Laurence Vass) had an SUV_max_ greater 1.5. This can likely be explained by the differences in experience between the two readers, with the first reader being trained as a general nuclear medicine physician and contouring abnormal attenuation correction CT findings. On the other hand, the second reader was specifically trained for ^18^F-FES PET contouring and therefore focused only on contouring high FES uptake lesions. This discrepancy highlights the difficulty of generating a reference standard dataset for PET imaging, because it is unclear whether models should be trained to detect abnormalities on CT images or only disease with high radiotracer avidity. Practically, the approach will depend on the desired application: if an estimate of radiotracer avidity is needed, low-uptake lesions can be left out of the reference standard dataset. Conversely, if it is desired to detect all abnormalities for an application such as computer-aided detection in generating patient reports, FES-negative disease may be included.

In the decision tree for determining whether a patient has FES-avid metastases, a patient was labeled as having (or likely having) FES-avid metastases if one (or more) lesions had an SUV_max_ greater than 1.5 (15). In participants with more than one lesion, the accuracy of this patient-level label is less dependent on accurately detecting a single lesion and can rely on simply finding any lesion. However, in 20 of the 52 participants, the automated status (of having FES-avid metastases or not) was determined by a single lesion (or absence of any lesion in the case of some participants). Therefore, the tool proved to be proficient (with a sensitivity of 90%) in automating the determination of whether participants had or did not have FES-avid metastases in participants with both high and low disease burdens. However, the high rate of false positives led to a low specificity (54.5%), which could be improved with a larger training set, enabling the model to better learn differences between healthy and pathologic tracer uptake.

There is a growing amount of literature investigating the impact of interlesion heterogeneity on therapy response and patient outcomes, showing that patients with FES-negative lesions show poorer response to endocrine therapy (33–35). One recent study (35) demonstrated the importance of measuring full-body heterogeneity for the prediction of response to endocrine therapy. However, depending on the disease burden, labeling all lesions from a PET/CT scan can take 50–60 minutes per case (17), which is infeasible in a clinical setting. The study by van Geel et al (35) showed that faster short-cut methods, such as a visual heterogeneity assessment and a "five largest lesion" method were not sufficient for prediction of response and that full-body lesion detection and concordance with ^18^F-FDG PET/CT were needed. Both tools presented in this study, for automated lesion detection and concordance analysis, are intended to facilitate full-body concordance analysis so that it can become clinically feasible. The automated lesion detection algorithm presented here takes between 1 and 3 minutes to run on a standard workstation, depending on the size of the field of view. Therefore, even with a review and confirmation of detected lesions, this tool has the ability to present significant time savings when doing a full-body heterogeneity assessment.

One pitfall of ^18^F-FES PET imaging is that any FES-avid liver metastases will be masked by the high physiologic uptake in the liver. For the lesion detection model, the accuracy of the model was calculated based on the reference standard read on the ^18^F-FES PET images. Therefore, no liver lesions were identified by the reader manually because liver lesions would also not be visible to the human eye. In the subset of images randomly chosen for the concordance analysis, only one patient had a lesion found on the standard-of-care images (^18^F-FDG PET/CT). This lesion was, as predicted, not identified at the ^18^F-FES PET/CT examination.

This study had numerous limitations. Due to limited dataset size as well as a lack of further imaging and follow-up, reasons for discordance presented in this study could not be further analyzed. Another limitation of the study was that, in this proof-of-concept work, the concordance analysis was performed using manually contoured lesions from each modality. The concordance analysis performed in this study is only achievable through automation; manual detection, segmentation, and matching of all lesions across modalities is infeasible in a clinical setting (35,36). The matching method used in this work has been shown to have performance similar to interphysician variability of the same task (17). A fully automated lesion detection algorithm for all modalities preceding an automated concordance assessment would provide the most comprehensive method for allowing quantification and characterization of all lesions in a patient with metastases. Although of interest for future work, this approach was not taken in the current study. In a clinical workflow, a fully automated approach may have important implications for assisting treatment decisions of both systemic and targeted therapies.

Other limitations that are important to note include that results from the automated lesion detection approach are exploratory and, although we performed cross-validation as a step toward ensuring model generalizability, our results do not represent performance in an external validation dataset. Further work is needed to gather larger datasets, determine optimal network architecture, and validate the tool across imaging centers. With larger datasets, free response operator curves could also be generated to adjust the operating point for higher sensitivity and lower specificity. This was not performed in the current study because the tuning dataset for each fold of cross-validation (two scans) was too small to determine an optimal point for application to the held-out data. Additionally, reference standard contours gathered from a consensus of experienced nuclear medicine physicians could improve the consistency of the training and testing data. This is apparent in an example ^18^F-FES PET/CT image that had 189 reference standard lesions, in which eight false positives were detected by the model. Of these eight false positives, five were located in the legs in high-uptake regions that could potentially be true positives missed by the physician due to contouring fatigue resulting from contouring disease in patients with such a high disease burden. A consensus contour via nuclear medicine physician panels would reduce the impact of physician fatigue in these high burden cases. Finally, although the Retina U-Net architecture was chosen to emphasize both lesion detection and segmentation during optimization, comparisons with the self-configuring nnU-Net (37), which has performed well in medical image segmentation tasks, are warranted as future work.

In conclusion, this work introduces an AI model that was trained to detect lesions on ^18^F-FES PET/CT images and an automated concordance tool that measured heterogeneity between ^18^F-FES PET/CT and standard-of-care imaging. Using these tools, we have demonstrated how an automated tool can be used to rapidly detect lesions in patients with metastatic breast cancer and determine whether a patient has ER-positive metastases. This determination can then be used to decide whether a patient is suitable for endocrine therapy. The automated concordance tool presented would allow clinicians to rapidly assess interlesion heterogeneity for a patient given more than one type of imaging. In future work, the plan is to link the two tools presented together by feeding the automatically detected lesions from both modalities (^18^F-FES PET/CT and ^18^F-FDG PET/CT or diagnostic CT) into the concordance tool. Additionally, in future studies, analyzing discordant results with respect to tumor subtype and prior treatments is needed. Further, correlating across multiple standard-of-care imaging modalities (eg, ^18^F-FES PET vs both CT and ^18^F-FDG PET) for a larger population should be performed.
